# Mutations Associated with Functional Disorder of Xanthine Oxidoreductase and Hereditary Xanthinuria in Humans

**DOI:** 10.3390/ijms131115475

**Published:** 2012-11-21

**Authors:** Kimiyoshi Ichida, Yoshihiro Amaya, Ken Okamoto, Takeshi Nishino

**Affiliations:** 1 Department of Pathophysiology, Tokyo University of Pharmacy and Life Sciences 1432-1, Horinouchi, Hachioji, Tokyo 192-0392, Japan; E-Mail: ichida@toyaku.ac.jp; 2 Division of Biochemistry, Niigata University Graduate School of Medical and Dental Sciences, 2-5274 Gakkocho-dori, Chuo-ku, Niigata 951-8514, Japan; E-Mail: amaya@dent.niigata-u.ac.jp; 3 Department of Biochemistry and Molecular Biology, Nippon Medical School, 1-1-5 Sendagi, Bunkyou-ku, Tokyo 113-8602, Japan; E-Mail: okamoto@nms.ac.jp; 4 Department of Applied Biological Chemistry, Graduate School of Agricultural and Life Sciences, University of Tokyo, 1-1-1 Yayoi, Bunkyo-Ku, Tokyo 113-8657, Japan

**Keywords:** xanthine dehydrogenase, xanthine oxidase, xanthine oxidoreductase, xanthine oxidoreductase deficiency, flavoproteins, xanthinuria, hereditary xanthinuria, gout

## Abstract

Xanthine oxidoreductase (XOR) catalyzes the conversion of hypoxanthine to xanthine and xanthine to uric acid with concomitant reduction of either NAD^+^ or O_2_. The enzyme is a target of drugs to treat hyperuricemia, gout and reactive oxygen-related diseases. Human diseases associated with genetically determined dysfunction of XOR are termed xanthinuria, because of the excretion of xanthine in urine. Xanthinuria is classified into two subtypes, type I and type II. Type I xanthinuria involves XOR deficiency due to genetic defect of XOR, whereas type II xanthinuria involves dual deficiency of XOR and aldehyde oxidase (AO, a molybdoflavo enzyme similar to XOR) due to genetic defect in the molybdenum cofactor sulfurase. Molybdenum cofactor deficiency is associated with triple deficiency of XOR, AO and sulfite oxidase, due to defective synthesis of molybdopterin, which is a precursor of molybdenum cofactor for all three enzymes. The present review focuses on mutation or chemical modification studies of mammalian XOR, as well as on XOR mutations identified in humans, aimed at understanding the reaction mechanism of XOR and the relevance of mutated XORs as models to estimate the possible side effects of clinical application of XOR inhibitors.

## 1. Introduction

**Figure 1:**
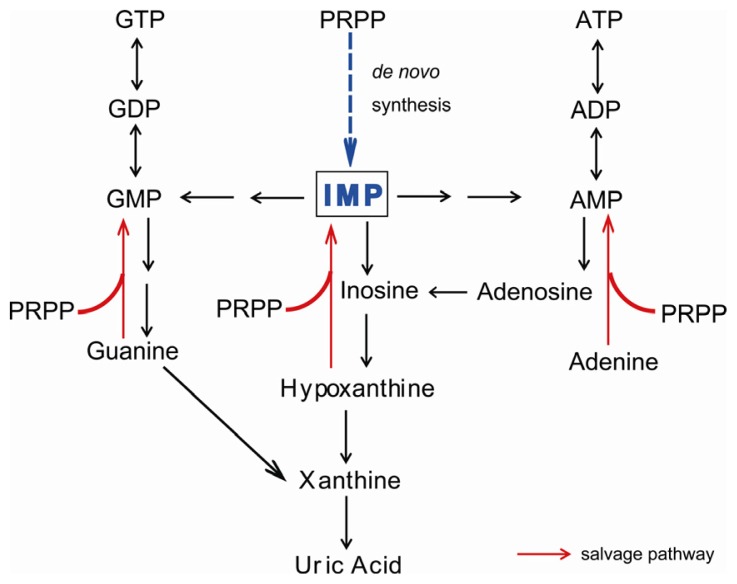
Metabolic pathways of purine degradation in humans. Xanthine oxidoreductase (XOR) catalyzes the transformations of hypoxanthine to xanthine and xanthine to uric acid. XOR-deficient patients secrete xanthine, which is formed from guanine. Accumulated hypoxanthine is mostly converted to inosine monophosphate (IMP) via the salvage pathway using 5-phospho-α-D-ribosyl 1-pyrophosphate (PRPP) as a co-substrate.

Xanthine oxidoreductase (XOR) catalyzes two hydroxylation steps in the metabolic pathway of purine degradation, *i.e.*, hypoxanthine to xanthine and xanthine to uric acid, utilizing either NAD^+^ or O_2_[1–3] (Figure 1). In higher animals, XOR exists as a homodimer of 150 kDa subunits [4]. Each subunit contains one molybdenum center (molybdenum cofactor; Moco), one flavin adenine dinucleotide (FAD) cofactor and two distinct iron sulfur centers ([2Fe-2S] type) [1–3]. The purine hydroxylation reaction occurs at the molybdenum center. Electrons, which are transferred to molybdenum during the hydroxylation reaction, are further transferred to FAD via the two iron sulfur centers [5,6]. Finally, NAD^+^ or oxygen molecule, which is the final electron acceptor, is reduced at the FAD center.

XOR has two forms: xanthine dehydrogenase (XDH), which prefers NAD^+^ as the substrate and xanthine oxidase (XO), which prefers O_2_[1]. Historically, XDH and XO have been studied as distinct enzymes. XOR has been isolated only as the XO form from mammalian sources, whereas it has always been purified in the XDH form from other organisms [2]. It is becoming clear, however, that mammalian XORs exist in the XDH form under normal conditions in the cell, but are converted to the XO form during extraction or purification, either irreversibly by proteolysis or reversibly by oxidation of cysteine residues to disulfide bridges. In some particular cases, XDH can be converted to the XO form [2]. The mechanism of conversion from XDH to XO has been thoroughly elucidated in the past decade by means of a range of techniques, including X-ray crystal structure analysis of various mutants, and it has become clear that the protein environment influences the reactivity of the FAD cofactor towards different substrates through substantial conformational changes triggered by modifications located far from the cofactor [5,6].

The enzyme is a target of drugs to treat hyperuricemia, gout or reactive oxygen-related diseases [7,8]. It is distributed in various organs including liver, mammary gland and endothelial cells of vascular vessels [9,10]. The enzyme was proposed to be localized in peroxisomes of rat liver [11], but was found to be present in cytosol [12]. As XOR inhibitors significantly lower uric acid production and concentration in the blood, they can be used to treat gout. Allopurinol, which was introduced by Elion *et al.*[13], has been on the market for over 40 years [14]. In recent years, however, several companies have developed very effective inhibitors [14–16], of which one example is febuxostat [17]. Clinical trials indicate that febuxostat is superior to allopurinol in lowering uric acid production, although the mechanism of inhibition is different [18,19]. By means of enzymatic, spectroscopic and structural-biological analyses of the inhibition mechanism, it has been shown that these recently developed inhibitors bind tightly to both the oxidized and reduced forms of XOR in a highly structure-specific manner [15], whereas allopurinol, a substrate analogue, binds covalently to the reduced molybdenum atom (MoIV) after having been converted to the hydroxylated product (oxipurinol: alloxanthine) [20], mimicking the reaction intermediate formed during the hydroxylation reaction with xanthine as a substrate [21]. Although oxipurinol binds very tightly to the enzyme, it can be dissociated from the molybdenum (VI) by spontaneous reoxidation due to electron transfer to other centers with a half-time of 300 min at 25 °C [20]. Potent inhibition seems to be essential to lower the uric acid level in blood or tissue, since XOR is a house-keeping enzyme that exists abundantly in various organs [10]. However, it has been suggested that lowering uric acid levels may cause side effects in humans, since uric acid acts as a radical scavenger in the body [22,23]. Further, it is proposed that NO formed by XOR via reduction of NO_2_ (with any electron donor) may induce vasodilatation under ischemic conditions [24–26]. On the other hand, XOR has the potential to generate oxygen radical species (H_2_O_2_ and O_2_^−^) after conversion from XDH to XO [1–6]. O_2_^−^ would rapidly react with NO to form ONOO^−^[27]. This reaction may serve to eliminate NO, at least in part, but the ONOO^−^ produced is highly toxic [28]. As to the question of potential NO formation by XOR, the activity for NO formation from NO_2_ is extremely low, even under anaerobic conditions, although from a chemical point of view it is possible that the water-exchangeable hydroxyl group at OH-Mo(IV) can be replaced by NO_2_ to produce NO, since various compounds, such as uric acid (which reacts very slowly to form xanthine), can behave similarly, as discussed by Okamoto [29]. The reported *k*_cat_ value of NO formation is 0.17 s^−1^ at 37 °C with NADH as an electron donor under anaerobic conditions [30], *i.e.*, less than 1% of *k*_cat_ for xanthine oxidizing activity (*k*_cat_ value 15–20 s^−1^ at 25 °C) [31,32]. It is questionable whether such a weak activity can have any physiological significance, even under ischemic conditions. The present review focuses mainly on mutational studies of XOR and mutations associated with hereditary dysfunction of XOR in humans, since these are useful for understanding the enzyme reaction mechanism and also as models to estimate the possible side effects of using XOR inhibitors as drugs.

## 2. Symptoms of XOR Deficiency and Differential Diagnosis

Human diseases associated with genetic dysfunction of XOR are termed xanthinuria, because xanthine is excreted in the urine [33]. Although the enzyme catalyzes two steps of reaction, as described above, so that XOR dysfunction might be expected to be associated with tissue accumulation of hypoxanthine due to inhibition of the first step (conversion of hypoxanthine to xanthine), in fact hypoxanthine is not normally significantly excreted in urine [34,35]. Instead, hypoxanthine is converted to inosine monophosphate (IMP) owing to activation of the salvage pathway (Figure 1) [36]. Patients typically have low levels of uric acid (less than 1 mg/dL) in blood, so XOR-deficient patients are frequently identified based on measurement of uric acid in blood. Various diseases or disorders other than xanthinuria may lead to hypouricemia (Table 1). Renal hypouricemia, which can be caused by decreased re-absorption due to impaired function of urate transporter in the nephrons, is also clinically asymptomatic in most cases.

Xanthinuria is classified into two subtypes, type I and type II (Table 1) [35]. The type I is due to a genetic defect of XOR, whereas the type II is due to a genetic defect in molybdenum cofactor sulfurase [37,38]. Aldehyde oxidase (AO), also a molybdoflavo enzyme, is similar to XOR. A terminal sulfide group is necessary as the third ligand in the active center of XOR and AO for enzymatic activation of these enzymes after biosynthesis of the molybdenum cofactor. Molybdenum cofactor sulfurase catalyzes this final maturation step by generating a protein-bound persulfide, which is the source of the terminal sulfur ligand of the molybdenum cofactor. Thus, lack of sulfurase results in type II xanthinuria. Type I and II xanthinuria are not clinically distinguishable. In order to differentiate them, allopurinol loading test and gene analysis are performed, because a measurement method for molybdenum cofactor sulfurase activity has not yet been established [39,40]. In the allopurinol loading test, oxipurinol is detected in serum and urine of type I xanthinuria patients after administration of allopurinol, as conversion of allopurinol to oxipurinol is catalyzed by XOR and AO, while oxipurinol is not detected in the case of type II xanthinuria. AO has broad substrate specificity, oxidizing different types of aldehydes and heterocyclic rings [41,42]. No clinical symptom or abnormal laboratory examination result due to lack of AO has yet been identified. However, it has recently been reported that AO plays an important role in the metabolism of numerous compounds. Thus, classification of type I and II xanthinuria might be indispensable for optimum medical treatment of patients with xanthinuria in the future.

In higher animals other than primates, xanthinuria is lethal due to kidney damage resulting from xanthine stones in the urinary tract [43–46]. Although primates have lost uricase during evolution and seem to have acquired tolerance to oxipurines, e.g., through downregulation of XOR gene expression, other animals convert uric acid to more soluble allantoin, catalyzed by peroxisomal uricase, and do not seem to have such tolerance [47]. Urolithiasis is sometimes accompanied with xanthinuria due to xanthine deposition, and rarely this may lead to acute renal failure [48–53]. In addition to its role in uric acid production, XOR has bactericidal activity via ROS generation under certain conditions, particularly in mammalian mammary gland [54]. In addition to the NO_2_ reduction as described in the previous section, XOR has been proposed to play a role in lactation, though the mechanism of the role in lactation remains unclear [55,56]. XOR has also been suggested to be implicated in hypertension, cardiovascular disorder, and adipogenesis [57,58]. Although the situation is not simple, clinical observations in xanthinuria patients with extremely low serum uric acid levels, who show no symptoms, suggest that administration of XOR inhibitors may not cause severe side effects if the inhibitor has no other effect than inhibition of XOR, except possibly in special cases, such as cancer, pregnant or breast-feeding patients. Many purine analogue cancer drugs, such as mercaptopurine, are known to be catabolized by XOR [59]. The third type of XOR deficiency, type III XOR deficiency, involves the molybdenum cofactor. Molybdenum cofactor deficiency involves triple deficiency of XOR, AO and SO (sulfite oxidase), due to a defect in the synthesis of molybdopterin, which is a precursor of molybdenum cofactor for all three enzymes. Symptoms of molybdenum cofactor deficiency include severe neurological disorder, lens dislocation and dysmorphism, and the outcome is poor [60].

## 3. Overall Structure of Human Xanthine Oxidoreductase (XOR)

The primary structure of human XOR was first reported by Ichida *et al.*[61], who isolated cDNA clones encoding human *XOR* by cross hybridization with rat cDNA, the structure of which was reported by Amaya in 1990 [62]. The *XOR* gene has 36 exons, and is located in chromosome 2p23.1 [63,64]. The primary structure of human XOR has 90% homology with the rat enzyme over the entire length. Although cloning of human XOR was subsequently reported by several groups [64–66], the sequences were all very similar, except for one reported by Wright et al that was later found to encode AO, not XOR. Although mammalian XORs and AOs from various sources have similar molecular weights and cofactors [2,6,67], their substrate specificities are different. AO exclusively utilizes O_2_ as the oxidizing substrate rather than NAD^+^, which is used by dehydrogenases. The specificities of the two enzymes for reducing substrates partially overlap, and each is capable of hydroxylating a distinct subset of a wide range of aldehydes and aromatic heterocycles. Purine bases are good substrates of XORs, but are not good substrates of AOs. The physiological substrates of mammalian AOs are not known, although the involvement of AOs in drug metabolism is well-established [42]. Structure-based sequence comparisons have identified residues in the vicinity of the active site molybdenum center of AO that differ from those in XOR, and these are most probably the determinants of the substrate preferences exhibited by the family members [68]. However, a glutamic acid residue, thought to represent an essential catalytic base, is strictly conserved in AOs and XORs, pointing to a common catalytic mechanism for all family members [69].

**Figure 2:**
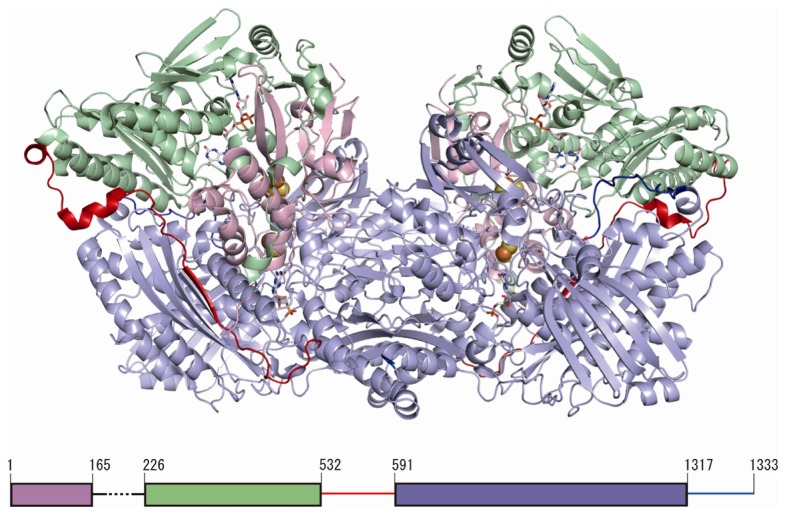
Structure of human XOR. The structure illustrated is that of a human mutant dimeric XDH [69] (PDB: 2E1Q). The Fe/S, FAD, and molybdopterin domains are colored light pink, light green and light blue, respectively. The interdomain loop (residues 533–590) is colored red. *C*-terminal is colored blue. A schematic representation of the domain structure in relation to the primary sequence is shown at the bottom.

The crystal structures of human XOR from natural milk at 3.6 Å resolution (PDB: 2CKJ) and recombinantly produced XDH at 2.6 Å resolution (PDB: 2E1Q) [69] are available. Higher resolution structures of mammalian XORs are available for native bovine XDH and XO, as well as recombinantly produced rat XDH and XO, including various mutants [69,70]. The subunits in the crystal structures of all these mammalian XORs are arranged as identical dimers that display a distinct butterfly shape [4,69,70]. The dimensions of the whole enzyme molecule are about 155 Å × 90 Å × 70 Å (Figure 2). Each monomer is composed of three subdomains. The small *N*-terminal domain (residues 1 to 165 in the human enzyme) contains both of the iron-sulfur centers (Fe/S I and Fe/S II) and is connected to the second, FAD-containing domain (residues 226 to 532, colored light green in Figure 1) via a long, partially disordered segment consisting of residues 166 to 225. The FAD domain, in turn, is connected to the third, *C*-terminal domain via another extended segment (residues 533 to 590), which is also partially disordered. The third and largest domain (residues 591 to 1317, colored light blue in Figure 2) binds Moco close to the interface of the Fe/S- and FAD-binding domains, connected with a *C*-terminal loop (residues 1318–1333, colored blue in Figure 2) [4,69,70].

## 4. Residues Crucial for Enzyme Function: Experimental Studies

In order to elucidate the mechanisms of hydroxylation at the molybdenum center, electron transfer within the redox centers and reoxidation of the reduced FAD by the natural substrate, NAD^+^ or molecular oxygen, various chemical modification and mutation studies have been performed during the last two decades. The amino acid residues of human XOR corresponding to those that have so far been found to be crucial for enzyme function, either by chemical modification or by mutagenesis studies with bovine or rat XORs, are summarized in Table 2.

### 4.1. The *N*-Terminal Fe/S Domain

This domain contains a cluster of two distinct [2Fe-2S] types, having different EPR signals and redox potentials, and these are named the Fe/S I and Fe/S II centers [77–79]. The Fe/S I signal displays *g*-values of *g*_1,2,3_ = 2.022, 1.932, 1.894, with line-widths and relaxation properties typical of a [2Fe-2S] cluster, while Fe/S II has g-values of *g*_1,2,3_ = 2.110, 1.991, 1.902, with unusually broad line widths and relaxation properties. The latter signals can only be observed below 25 K [80]. Site-directed mutagenesis studies employing heterologously expressed rat XOR have allowed assignment of the two distinct types of EPRsignals to the respective clusters [71], with Fe/S I being located in the unusual-^113^Cys-Xaa_2_-^116^Cys-//-^148^Cys-Xaa_1_-^15^ Cys-motif in the α-helical domain and Fe/S II in the *N*-terminal-^43^Cys-X-^48^Cys-X-^51^Cys-//-^73^Cys-motif in the ferredoxin-like domain. This establishes the sequence of electron transfer within the enzyme molecule as Mo → Fe/S I → Fe/S II → FAD. It was noted that the mutation at Cys43Ser or Cys51Ala, which are both components of Fe/S I, resulted in the appearance of insoluble or monomeric proteins, suggesting the importance of the Fe/S I cluster for protein conformation and/or folding [71].

### 4.2. The Intermediate FAD Domain

**Figure 3:**
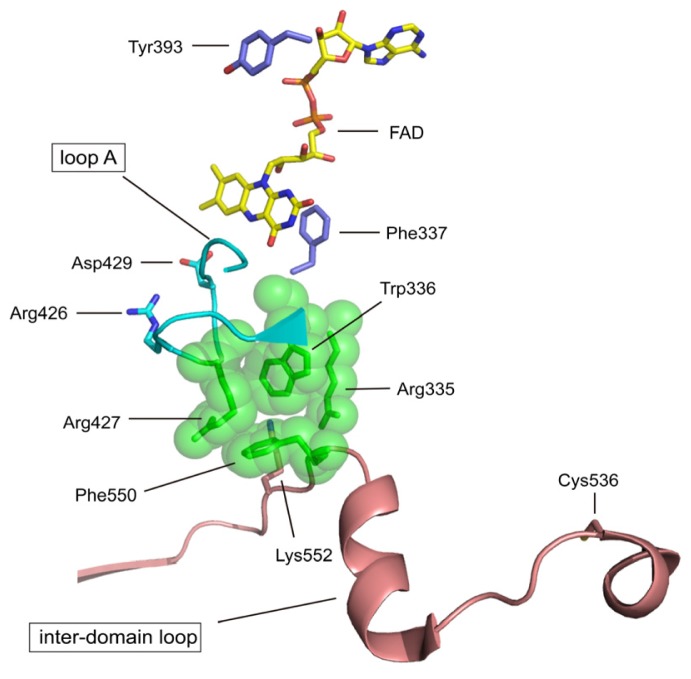
Structure of the active site cavity of FAD in human XOR. FAD is shown as a yellow colored stick model. The amino acid residues experimentally studied with various systems are listed in Table 2. The unique amino acid cluster consisting of the side chains of Arg427, Arg335, Trp336 and Phe550, is shown as a space-filling model in green (PDB: 2E1Q).

The domain binds its cofactor FAD in a deep cleft; in the NAD-free form, the *si*-face of the isoalloxazine ring is exposed to solvent (Figure 3). The same space allows the substrate NAD access to the flavin, and the two ring systems stack on top of each other [81]. Modification of the chicken XDH residue corresponding to Tyr419 (human Tyr393) with fluorosulfonylbenzoyl adenosine (FSBA) resulted in loss of activity towards NAD^+^[73], suggesting that this tyrosine residue is indeed involved in NADH binding, as indicated by the crystal structure of the rat XDH-NADH complex (Tomoko Nishino, K. Okamoto, E.F. Pai and Takeshi Nishino, unpublished data). In contrast to the open *si*-side, the *re*-side of the flavin ring is in tight contact with residues of the protein chain, e.g., the side chain of Phe336 (human Phe337) lies parallel to the isoalloxazine ring. Mutation study indicated that this phenyl-flavin pair may serve to tune the cofactor’s FAD redox potential (Tomoko Nishino, K. Okamoto, E.F. Pai and Takeshi Nishino, unpublished data). In the crystal structures of XDH and XO, the location of so-called loop A (residues 423–433 in human XOR) is very different in rat and bovine XORs. In rat and bovine XDH, the side chain of Asp428 (rat sequence, corresponding to human & bovine Asp429) in the loop is close to C_6_ of the flavin. This residue must be a major contributor to the strong negative charge at the flavin-binding site [4,81]. Mutation of this residue with rat XOR changes the reactivity of FAD by changing its redox potential (Y. Kawaguchi *et al.* unpublished). In XO conformation, Asp428 moves away from the flavin ring and the guanidinium group of Arg425 replaces it, approaching the nearest atom of the isoalloxazine ring to within 6.3 Å. This reversal of the electrostatic potential surrounding the redox-active part of the FAD cofactor matches predictions based on biochemical and biophysical studies of the XDH and XO forms [82–84]. Bovine Arg427, Arg335, Trp336 and Phe549 (human Arg335, Trp336, Arg427 and Phe550) are components of a unique cluster of four amino acids [72], which are held together mostly via π-cation interactions in the XDH form. Phe549 (rat Phe549, human Phe550) is located in the long linker between the intermediate FAD and *C*-terminal Moco domains. In the XO form, however, this cluster is disrupted (Figure 3) [5]. An equivalent effect can be achieved by mutating one of these residues with rat XOR [72]. Proteolysis at Lys551 (human Lys552) [62], leading to drastically increased mobility of the linker peptide between the intermediate FAD and *C*-terminal Moco domains, or disulfide formation between Cys535 and Cys992 [70,74], causing conformational strain, breaks Phe549 out of this tight arrangement. Disruption of the cluster is accompanied with movement of the active site loop A. Recent studies suggest that the conversion from XDH to XO is in equilibrium [85]; the highly packed amino acid cluster, binding of NAD^+^/NADH and insertion of the *C*-terminal peptide shift the equilibrium towards the XDH form, while disulfide formation between Cys535 and Cys992 (human Cys536 and Cys993) or proteolysis in the linker between the FAD and the Moco domains disrupts the amino acid cluster and moves the active site loop A. In rat enzyme extrusion of the C-terminal peptide, by formation of a disulfide bond between Cys1316 and Cys1324, shifts the equilibrium partially to the XO form.

### 4.3. The *C*-Terminal Moco Domain

**Figure 4:**
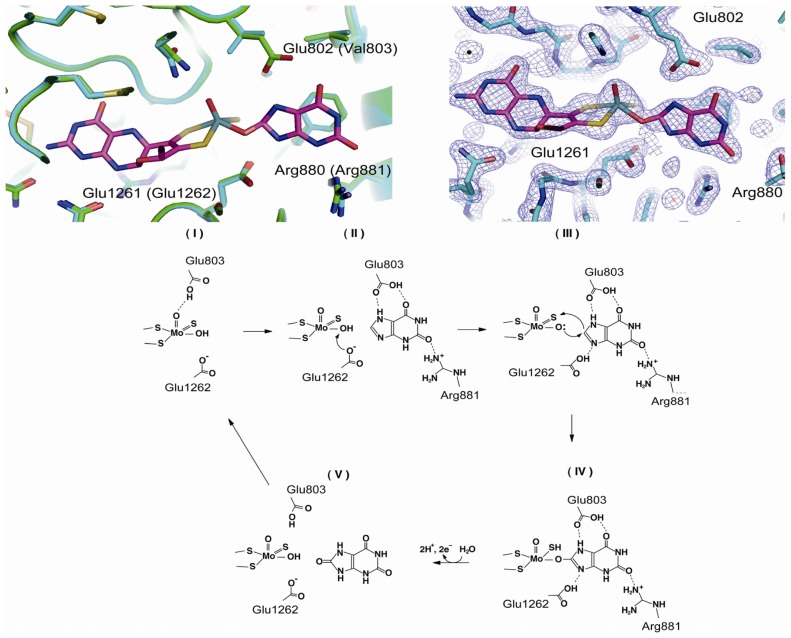
Binding modes of the substrate xanthine and mechanism of its hydroxylation. Upper left, superposition of the two crystal structures around Moco of human E803V mutant XDH (cyan) and reduced native bovine XDH in the urate-bound form (green) [86]. Upper right, electron-density map of reduced native bovine XDH with bound urate [86] (PDB: 3AMZ). Lower, proposed hydroxylation mechanism based on the crystal structure of the urate-bound form and the results of mutation studies [69].

The large third domain (residues 591 to 1317, colored light blue in Figure 2) sequesters Moco close to the interface of the Fe/S- and FAD-binding domains. However, recombinantly expressed proteins, including human and rat enzymes, lack Moco, likely due to overloading of the Moco synthesis and insertion enzymes in the expression system [69,70]. High-resolution crystal structure analysis of a mutant of rat XDH (1.7 Å) indicated that the conformation of the polypeptide chain surrounding Moco is very similar to that found in the native bovine milk enzyme [86]. Although the amino acid residues in the active site do not differ greatly in their positions and orientations, crystallographic information regarding the interactions of amino acid residues with substrates and inhibitors is based only on data for native fully active bovine XOR, and the mechanism of hydroxylation has been well understood only in the last decade. The amino acid residues directly involved in substrate binding and catalysis are Glu803, Arg881 and Glu1262 (human sequence) [69] (Figure 4). In the oxidized form of XORs, the Mo ion is in the +VI oxidation state, surrounded by an oxo– (=O) at the apical position, and one hydroxo (–OH) and one sulfido (=S) ligand in the equatorial plane [16], in addition to the two vicinal sulfur ligands contributed by the pterin group (Figure 4). It is now generally accepted that XOR transfers the –OH to the substrate [6,76] (Figure 4). Proton transfer occurs upon substrate binding from Mo-OH to Glu1262, and the protonated Glu1262 forms a hydrogen bond with substrate nitrogen atom, facilitating nucleophilic attack on the adjoining carbon by the oxygen atom, which has become a base (Mo-O^−^) [6,76]. When the residue corresponding to Glu1262 was mutated, the enzyme was completely inactivated [69,87]. Regarding the activating role of the charged residues of the active center, it was found that purine hydroxylation activity is significantly decreased by mutation of two residues, Glu803 and Arg881, in the active site cavity of human XOR into the corresponding residues in the amino acid sequence of AO, Val803 and Met881, respectively [69]. However, the mutants exhibited significant AO activity. Proposed binding modes of substrates hypoxanthine and xanthine (Figure 4) have been proposed based on kinetic analysis of mutants, as illustrated in Figure 4. Those binding modes suggest that the activation mechanism facilitates nucleophilic reaction through hydrogen bond formation between the substrate and amino acid residues (Figure 4 bottom). The interaction of the 2-position keto group (C=O) and Arg881 is crucial for the efficacy of hydroxylation of the 8-position. These mechanisms are consistent with the metabolic sequence that hydroxylation of the 2-position of hypoxanthine precedes that at the 8-position [88–90]. X-Ray crystallography of the urate-bound reduced bovine XDH having full activity is consistent with this binding mode [86], as are the results of QM/MM studies with bovine XOR [91]. It was reported that two lysine residues were modified with fluorodinitrobenzene (FDNB) at pH 8.5, resulting in a decrease of activity due to slower release of the product, urate [75]. These residues were identified as Lys754 and Lys771 with rat XOR [74], both of which are located near the surface of the Moco domain, which may explain their accessibility to this chemical reagent. One of the nitro groups of DNB incorporated into a lysine residue of the enzyme was reported to be converted to an amino group due to reduction by substrate xanthine; this residue is most likely Lys771, which is rather close to the active site of the molybdenum center. Possible mechanisms will be discussed below.

## 5. Mutations Causing Type I Xanthinuria

Although inherited XOR deficiency was first reported in 1954 [33], detailed analysis of mutation sites of XOR was first reported in 1997 [37], and subsequently there have been several reports on XOR protein mutations associated with xanthinuria, as summarized in Table 3, including recent work on SNPs not necessarily associated with xanthinuria. The incidence of XOR deficiency, including type II, has been reported to be 1/69,000, but SNP analysis suggested a higher frequency of mutation in XOR, possibly because most mutations not cause dysfunction, being asymptomatic or merely producing a lower level of uric acid in blood.

Any mutation that causes nonsense substitution [92,94–96,98,99] can be expected to cause loss of activity, since the active site of xanthine hydroxylation lies in the *C*-terminal domain and therefore truncated proteins should be inactive for hydroxylation. Arg881X is the longest peptide among the reported mutants having a stop codon (Table 3), and as the stop codon site is just at the active site region, as described above, it seems very likely that an active site cavity cannot be formed.

The mutation of Arg149Cys at the Fe/S I cluster motif [93] may influence the formation of the cluster, resulting in loss of electron transfer, even if the protein is completely processed and folded. Thr910 is located at a distance of 7.3 Å from Mo=S in the molybdenum center. Mutation of this residue to a bulky methionine or lysine residue seems likely to result in the loss of Moco or its sulfur atom, which is essential for the activity. Alternatively, insertion of the lysine residue may change the electrostatic environment in the active center cavity.

SNP analysis suggests that mutations of XOR may be quite frequent [97]. Although the conditions of activity determination, such as XDH/XO ratio and content of the desulfo-form of each mutant may have varied, it was reported that mutation of some residues not directly involved in the catalysis may result in partial loss of activity, possibly through effects on the protein conformation. It is intriguing to note that mutants Ile703Val and His1221Arg show increased activity due to an increase of *V*_max_. Those residues are located not in the active site cavity, but rather at the surface of the *C*-terminal Moco domain. As stopped-flow studies with XDH showed that the rate-limiting step of the overall reaction is release of urate, such mutation might increase the rate of release of urate. It has been reported that the *k*_cat_ value of bacterial XDH is 10 times higher [87] and the enzyme inhibition pattern is very different from that of mammalian enzyme, *i.e.*, bacterial XDH was not efficiently inhibited by febuxostat, a potent inhibitor of the mammalian enzyme [100]. Molecular dynamic simulation indicated that the bacterial enzyme molecule is much more mobile due to different mobility of surface amino acid residues, suggesting that the release rate of urate may be slower than that of the mammalian enzyme. This may be consistent with the finding that the modification of surface amino acid residues with FDNB caused slower release of urate, as described in the previous section.

## 6. Type II Xanthinuria Is the Consequence of Mutation of Human Moco Sulfurase Gene

As described above, the molybdenum atom of XOR and AO is coordinated by 5 atoms, of which one is a sulfide atom (Mo=S). During hydroxylation, two electrons from the substrate are transferred as hydride to Mo=S to form Mo-SH. The natural preparation is known to contain a significant amount of inactive form in which the sulfide atom (S) is replaced by an oxygen atom (O) [101]. The ratio Mo=O/Mo=S varies from batch to batch. The enzyme can be inactivated spontaneously by loss of sulfide, and the sulfide can also be removed by CN treatment to give SCN [101,102]. Fully active enzyme (Mo=S) can be separated using affinity chromatography [103,104]. The amount of desulfo-form seems to be regulated by the sulfur-donating activity in various organisms, including fly [102] or chicken [105,106], suggesting the existence of a sulfur-donating enzyme, Moco sulfurase. In *Drosophila melanogaster*, some mutations at maroon-like locus (*ma-l*) are known to cause inactivation of both XDH and XO [107], and combined deficiency of XOR and AO in humans was reported [108]. In 1995, it was proposed that type II xanthinuria might be due to a defect in sulfur donation, resulting in combined deficiency of XOR and AO [35]. Subsequently the *ma*-*l* gene, bovine Moco sulfurase gene and finally human Moco sulfurase gene were cloned and sequenced; all of them are members of a superfamily having a *NifS*-like domain in the *N*-terminal followed by a possible Moco-binding domain with a total of 888 amino acids [38,109,110]. Two independent xanthinuria patients were found to having a mutation that converts codon 419 to a nonsense codon [38]. Subsequently, other mutants, Ala156 to Pro [111] and Arg776 to Cys [112], were reported to cause type II xanthinuria. As human Moco sulfurase has not yet been successfully expressed as a soluble protein and its three-dimensional structure is not available, we can only speculate that the mutations cause some conformational change or folding error that affects Moco binding. Further studies can be expected on this interesting protein and on the mechanism of sulfur incorporation, including the question of whether the sulfur atom is incorporated before or after Moco is incorporated into XOR or AO protein.

## Figures and Tables

**Table 1:** Causes of hypouricemia.

**Inherited disorders of purine metabolism**

Genetic defects in the molybdoflavoprotein enzymes:
Xanthinuria type I (xanthine oxidoreductase deficiency)
Xanthinuria type II (molybdenum cofactor sulfurase deficiency: combined xanthine oxidoreductase and aldehyde oxidase deficiencies)
Molybdenum cofactor deficiency
Purine nucleoside phosphorylase deficiency
Phosphoribosylpyrophosphate synthetase deficiency

**Secondary reduction in uric acid biosynthesis**

Hepatic failure

**Inherited renal hypouricemia (isolated renal tubule reabsorption defect)**

Renal hypouricemia-1 [URAT1 (SLC22A12) deficiency]
Renal hypouricemia-2 [URAT9 (SLC22A9) deficiency]

**Inherited causes of the Fanconi renotubular syndrome and its variants (the syndrome of multiple renal tubule reabsorption defects)**

Fanconi renotubular syndrome 1
Cystinosis (accumulation of intralysosomal cystine)
Galactosemia (galactose-1-phosphate uridylyltransferase deficiency)
Hereditary fructose intolerance (fructose 1-phosphate aldolase B deficiency)
Glycogen storage disease type 1 (glucose-6-phosphate deficiency)
Wilson’s disease [ATPase, Cu^2+^ transporting, beta polypeptide (ATP7B) deficiency]
Mitochondrial complex IV deficiency (cytochrome c oxidase deficiency)

**Acquired causes of the Fanconi renotubular syndrome and its variants**

Metal poisoning (e.g., Cd, Zn, Cu, Pb, Hg)
Multiple myeloma
Nephrotic syndrome
Malignant disease
Autoimmune disease (e.g., Sjogren’s syndrome)
Thermal burns
Primary hyperparathyroidism
Acute renal tubular necrosis
Renal transplant rejection

**Drugs**

Xanthine oxidoreductase inhibitor (e.g., allopurinol, febuxostat)
Drugs used either as uricosuric agents or to block other aspects of renal tubule excretion (e.g., sulfinpyrazone, probenecid, benzbromarone)
Non-steroidal anti-inflammatory drugs with uricosuric properties (e.g., phenylbutazone, azapropazone, high dose of aspirin)
Coumarin anticoagulants (e.g., warfarin)
Outdated tetracycline (5 alpha-6-anhydro-4-epitetracycline)

**Nutritional deficiencies**

Vitamines B_12_, C, D
Kwashiorkor

**Table 2:** Residues crucial for enzyme function revealed by experimental studies.

Corresponding human residue No.	Residue in experimental animal	Function	Experiments
**The Fe/S domain**			

Cys43	rat Cys43	Fe/S II ligand	mutation to Ser [71]
Cys51	rat Cys51	Fe/S II ligand	mutation to Ser or Ala [71]
Cys116	rat Cys115	Fe/S I ligand	mutation to Ser [71]
Lys185	rat Lys184	interdomain	Trypsin [62]

**The FAD domain**			

Arg427	bovine Arg427	A member of the cluster XDH/XO conversion	mutation to Gln [72]
Arg335	bovine Arg335	A member of the cluster XDH/XO conversion	mutation to Ala [72]
Trp336	bovine Trp336 & rat Trp335	A member of the cluster XDH/XO conversion	mutation to Ala [72]
Phe337	rat Phe336	redox potential of FAD	mutation to Leu (to be published)
Tyr393	chicken Tyr419	NAD^+^ binding	chemical modification with FSBA [73]
Asp429	rat Asp428	redox potential of FAD	mutation (to be published)
Cys536	rat Cys535	disulfide formation with Cys992 XDH/XO conversion	mutation to Ala [70] & chemical modification with FDNB [74]
Lys552	rat Lys551	Interdomain trypsin XDH/XO	Trypsin [62]

**The Moco domain**			

Lys755	bovine Lys754	*k*_cat_ slower	chemical modification with FDNB [74,75]
Lys772	bovine Lys771	*k*_cat_ slower	chemical modification with FDNB [74,75]
Glu803	human	purine binding	mutation to Val [69]
Arg881	human	purine binding	mutation to Met [69]
Cys993	rat Cys992	disulfide with Cys535 XDH/XO conversion	mutation to Arg [70] & chemical modification with FDNB [74]
Glu1262	human		mutation to Ala [69,76]
Cys1318	rat Cys1316	disulfie with Cys1324?	mutation to Ser [70]
Cys1326	rat Cys1324	disulfide with Cys1316?	mutation to Ser [70] & chemical modification with FDNB [74]

**Table 3:** Mutants causing type I xanthinuria.

Codon change	Amino acid change	Codon number	Phenotype	Reference
c. 140_141insG (c. 140dupG)	p.Cys48LeufsX12	47	Xanthinuria, type 1	[92]
c. 445C > T	p.Arg149Cys	149	Xanthinuria, type 1	[93]
c. 641delC	p.Pro214GlnfsX4	214	Xanthinuria, type 1	[94,95]
c. 682C > T	p.Arg228X	228	Xanthinuria, type 1	[37]
c. 1664_1665insC (c.1664dupC)	p.Ala556SerfsX15	555	Xanthinuria, type 1	[96]
c. 1663C > T	p.Pro555Ser	555	Decreased activity	[97]
c. 1820G > A	p.Arg607Gln	607	Decreased activity	[97]
c. 1868C > T	p.Thr623Ile	623	Decreased activity	[97]
c. 2107A > G	p.Ile703Val	703	Increased activity	[97]
c. 2164A > T	p.Lys722X	722	Xanthinuria, type 1	[98]
c. 2473C > T	p.Arg825X	825	Xanthinuria, type 1	[95]
c. 2567delC	p.Thr856LysfsX73	856	Xanthinuria, type 1	[37,96]
c. 2641C > T	p.Arg881X	881	Xanthinuria, type 1	[95]
c. 2727C > A	p.Asn909Lys	909	Decreased activity	[97]
c. 2729C > A	p.Thr910Lys	910	XDH deficiency	[97]
c. 2729C > T	p.Thr910Met	910	Xanthinuria, type 1	[52,92]
c. 3449C > G	p.Pro1150Arg	1150	Decreased activity	[97]
c. 3662A > G	p.His1221Arg	1221	Increased activity	[97]
c. 3953G > A	p.Cys1318Tyr	1318	Decreased activity	[97]
